# Neuroprotective Activity of Enantiomers of Salsolinol and *N*-Methyl-(*R*)-salsolinol: In Vitro and In Silico Studies

**DOI:** 10.1021/acsomega.3c05527

**Published:** 2023-10-05

**Authors:** Magdalena Kurnik-Łucka, Gniewomir Latacz, Adam Bucki, Mario Rivera-Meza, Nadia Khan, Jahnobi Konwar, Kamil Skowron, Marcin Kołaczkowski, Krzysztof Gil

**Affiliations:** † Department of Pathophysiology, Jagiellonian University Medical College, 31-008 Krakow, Poland; ‡ Department of Technology and Biotechnology of Drugs, Jagiellonian University Medical College, 31-008 Krakow, Poland; § Department of Medicinal Chemistry, Jagiellonian University Medical College, 31-008 Krakow, Poland; ∥ Laboratory of Experimental Pharmacology, Faculty of Chemical Sciences and Pharmaceutical Sciences, University of Chile, 8380494 Santiago, Chile

## Abstract

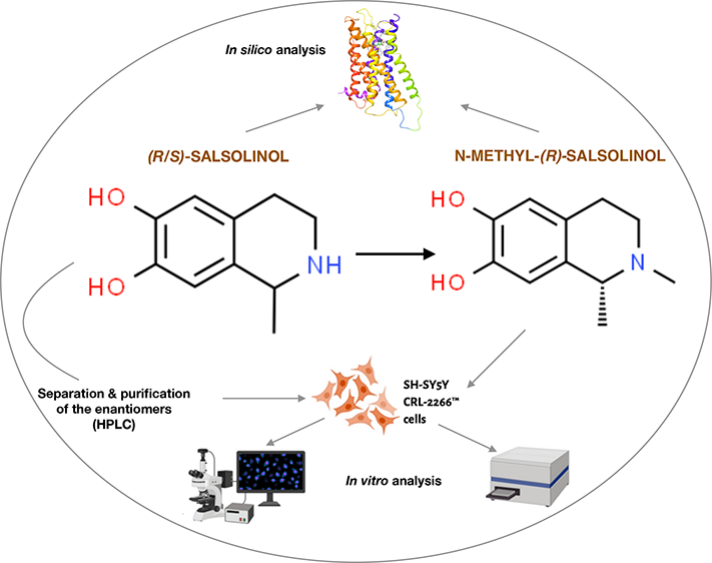

Salsolinol (1-methyl-1,2,3,4-tetrahydroisoquinoline-6,7-diol) is a close structural analogue of dopamine with an asymmetric center at the C1 position, and its presence in vivo, both in humans and rodents, has already been proven. Yet, given the fact that salsolinol colocalizes with dopamine-rich regions and was first detected in the urine of Parkinson’s disease patients, its direct role in the process of neurodegeneration has been proposed. Here, we report that *R* and *S* enantiomers of salsolinol, which we purified from commercially available racemic mixture by means of high-performance liquid chromatography, exhibited neuroprotective properties (at the concentration of 50 μM) toward the human dopaminergic SH-SY5Y neuroblastoma cell line. Furthermore, within the study, we observed no toxic effect of *N*-methyl-(*R*)-salsolinol on SH-SY5Y neuroblastoma cells up to the concentration of 750 μM, either. Additionally, our molecular docking analysis showed that enantiomers of salsolinol should exhibit a distinct ability to interact with dopamine D2 receptors. Thus, we postulate that our results highlight the need to acknowledge salsolinol as an active dopamine metabolite and to further explore the neuroregulatory role of enantiomers of salsolinol.

## Introduction

Up to date, it has been suggested that environmental and lifestyle factors together with a genetic profile determine if someone will develop Parkinson’s disease (PD), a neurodegenerative disorder with a broad spectrum of motor and nonmotor features.^1^ Although some progress has been made in the understanding of PD etiopathogenesis, currently no modifying therapies exist that successfully delay the progression of the disease. Levodopa remains the gold standard in the symptomatic treatment of PD as the majority of patients usually require levodopa therapy within two years of the onset of typical motor symptoms, yet, at the same time, about one-third of those patients experience dyskinesia and dystonia within two years after an initiation of the therapy.^2,3^ Whether an increased dopamine (DA) concentration or DA dyshomeostasis in the brain is entirely responsible for those side effects remains hypothetical^4^ and no compelling evidence that postponed initiation of levodopa treatment delays the onset of dyskinesia exists.^2^

A close structural analogue of DA, namely, salsolinol (SAL, IUPAC name: 1-methyl-1,2,3,4-tetrahydroisoquinoline-6,7-diol), whose presence in humans was first detected in the urine of PD patients treated with levodopa,^5^ colocalizes with DA-rich regions in the brain. SAL is a member of tetraisoquinolines family with one asymmetric center at C-1 and thus occurs as two stereoisomers heterogeneously distributed across the human brain.^6−8^ Whether SAL enantiomers decrease or increase in PD brain remains very uncertain.^9,10^ For example, racemic SAL was identified in the cerebrospinal fluid (CSF) of PD patients, but not in the control samples,^11^ and urine racemic SAL levels were the highest in PD patients with hallucinations.^12^ Racemic SAL concentrations were also significantly increased in the lumbar CSF of PD patients with dementia, regardless of PD degree or levodopa dosage,^13^ and furthermore, an elevated level of racemic SAL in CSF was proposed to be an indicator of the advancement of the disease.^14^ Yet, it was also reported that levels of both (*R*)- and (*S*)-SAL in untreated de novo PD patients were indifferent from matched healthy controls.^15^ Endogenously, racemic SAL is a product of nonenzymatic condensation of DA with acetaldehyde,^16^ while (*R*)-SAL should result from stereoselective enzymatic synthesis via (*R*)-salsolinol synthase.^17,18^ Yet, whether a loss of DA neurons and/or impaired DA metabolism could alter SAL concentration in the brain, or vice versa, remains unknown.

The concept of SAL contribution to the pathogenesis of idiopathic PD has emerged from the consideration of its chemical similarity to MPTP (1-methyl-4-phenyl-1,2,3,6-tetrahydropyridine), a well recognized neurotoxin,^12^ and further from the formation of its metabolites such as *N*-methyl-(*R*)-salsolinol (NMSAL) and 1,2-dimethyl-6,7-dihydroxyisoquinolinium ions.^17,19^ NMSAL, which was reported to be neurotoxic to dopaminergic nigrostriatal neurons in vitro, tends to decrease in CSF of PD patients with the disease progression.^20^ Still, SAL due to the presence of the catechol (1,2-dihydroxybenzene) moiety could possess antioxidant/neuroprotective properties.^21,22^ And indeed, we already reported that low doses of racemic SAL exhibited neuroprotective properties in SH-SY5Y cell line,^23,24^ which remains in accordance with some previous suggestions.^20^ SAL as a DA-derivative, could also target multiple binding sites, reviewed in ref (25), which is exemplified by inconclusive yet factual investigations.^26−29^ Clearly, an application of molecular modeling studies should also supplement our ongoing search for its mechanism of action and structure–activity relationships. Thus, the main aim of our study was 2-fold: (1) to assess in vitro toxicity of SAL enantiomers and (2) to model their ability to bind to dopamine D2 receptors via molecular docking analyses. Moreover, the in vitro toxicity and/or neuroprotection of commercially available NMSAL together with its ability to bind to dopamine D2 receptors via molecular docking analyses were evaluated.

## Results

### In Vitro Experiments

**Figure 1:**
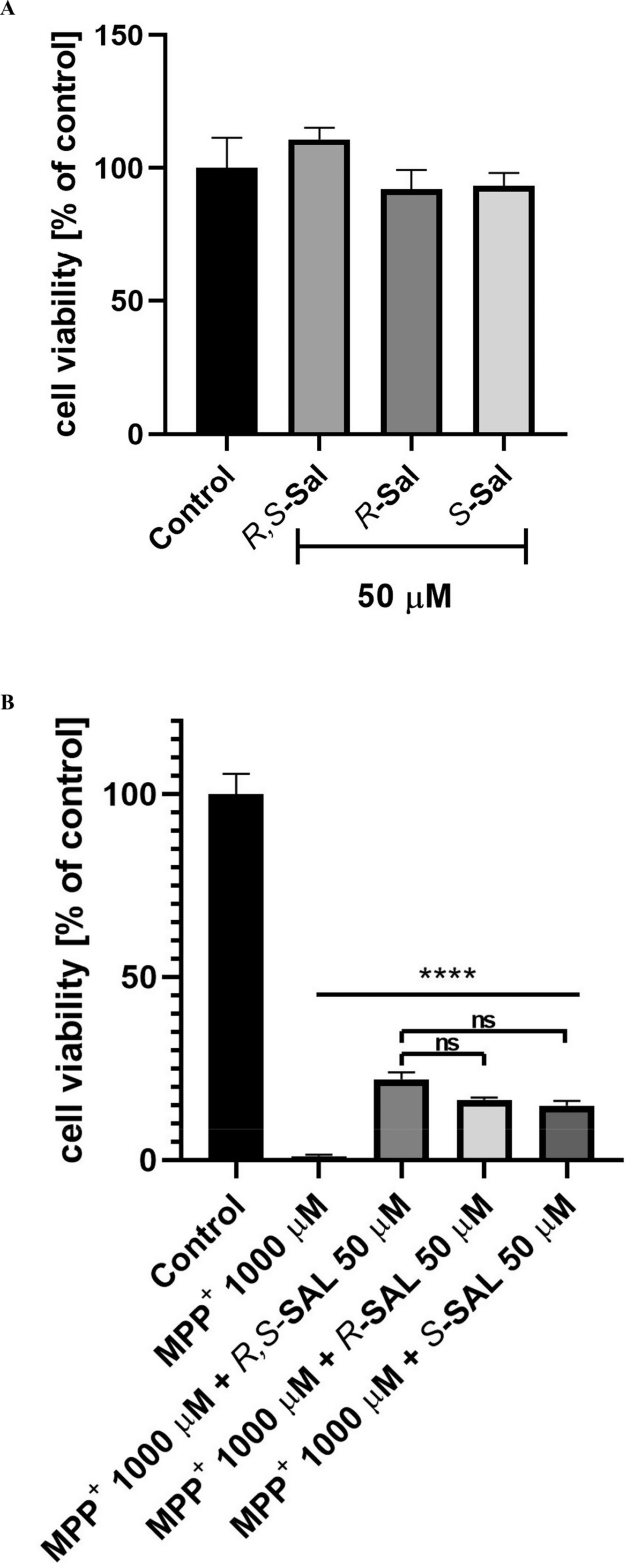
(A) Results of MTS test showing no toxic effect of 50 μM (*R*), (*S*), and (*R,S*)-SAL on SH-SY5Y neuroblastoma cells viability. Modified Eagle’s medium with 10% fetal bovine serum was used as the control. (B) Results of the MTS test showing the neuroprotective effect of 50 μM (*R*), (*S*), and (*R,S*)-SAL on SH-SY5Y neuroblastoma cells viability damaged by 1000 μM of MPP^+^ after 48 h of incubation. Modified Eagle’s medium with 10% fetal bovine serum was used as the control. Statistical significance was set at ^∗∗∗∗^*p* < 0.001 in comparison with the positive control: 1000 μM MPP^+^. No significant (ns) difference in the neuroprotective potency between racemate and the enantiomers was observed.

**Figure 2:**
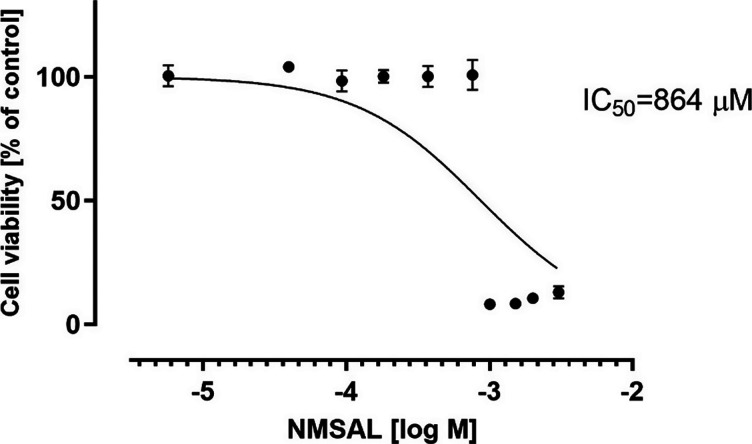
Results of MTS test showing no toxic effect of NMSAL on SH-SY5Y neuroblastoma cell viability up to 750 μM after 48 h of incubation. IC_50_ was calculated by GraphPad Prism 8.0.

**Figure 3:**
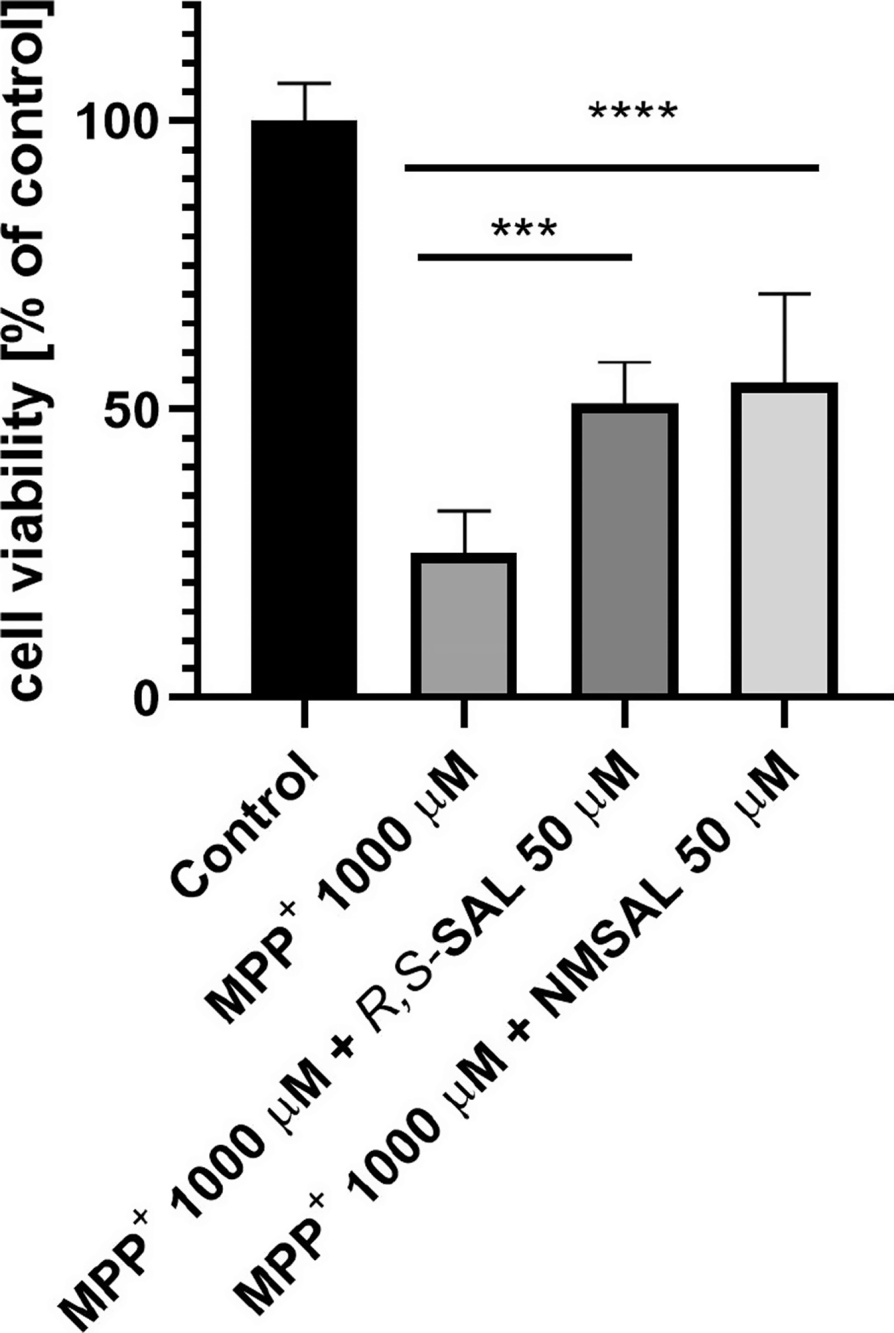
Results of MTS test showing the neuroprotective effect of 50 μM NMSAL and the reference (*R,S*)-SAL on SH-SY5Y neuroblastoma cells viability damaged by 1000 μM of MPP^+^ after 48 h of incubation. Modified Eagle’s medium with 10% fetal bovine serum was used as the control. Statistical significance was set at ***p > 0.001 and ****p < 0.0001 in comparison with the positive control: 1000 μM MPP^+^.

MTS assay showed (Figure 1) no toxic effect of 50 μM (*R*), (*S*), and (*R,S*)-SAL on human SH-SY5Y neuroblastoma cells viability. NMSAL also showed no toxicity up to 750 μM and the IC_50_ (half-maximal inhibitory concentration) was 864 μM (Figures 2 and 3). The statistically significant (*p* < 0.001) increase in viability of SH-SY5Y cells treated with MPP^+^ (1000 μM) and either racemic SAL or its enantiomers (all in the dose of 50 μM; Figure 1B) was observed in comparison with cells treated with MPP^+^ (1000 μM) alone (positive control). Furthermore, the MTS assay showed the neuroprotective effect of 50 μM NMSAL and the reference racemic SAL on SH-SY5Y cell viability damaged by 1000 μM MPP^+^ after 48 h of incubation (Figure 3).

**Figure 4:**
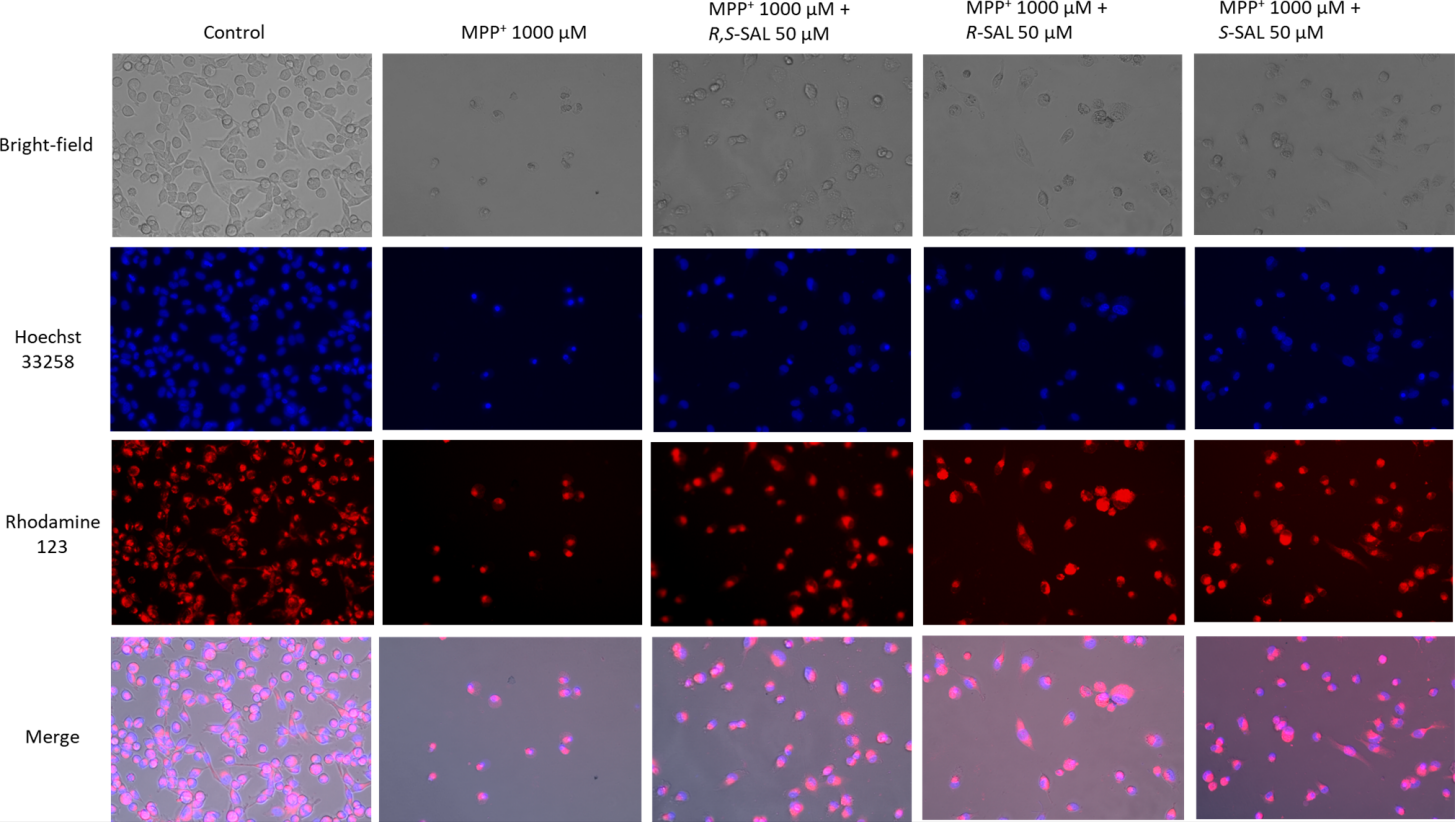
Microscopy images of SH-SY5Y cells exposed for 48 h on 1000 μM MPP^+^ and 1000 μM MPP^+^ together with 50 μM (*R*), (*S*), and (*R,S*)-SAL (all in 50 μM). Modified Eagle’s medium with 10% fetal bovine serum was used as the control. Representative pictures were taken by a Leica DMi8 fluorescence microscope Leica DMi8 (200×).

**Figure 5:**
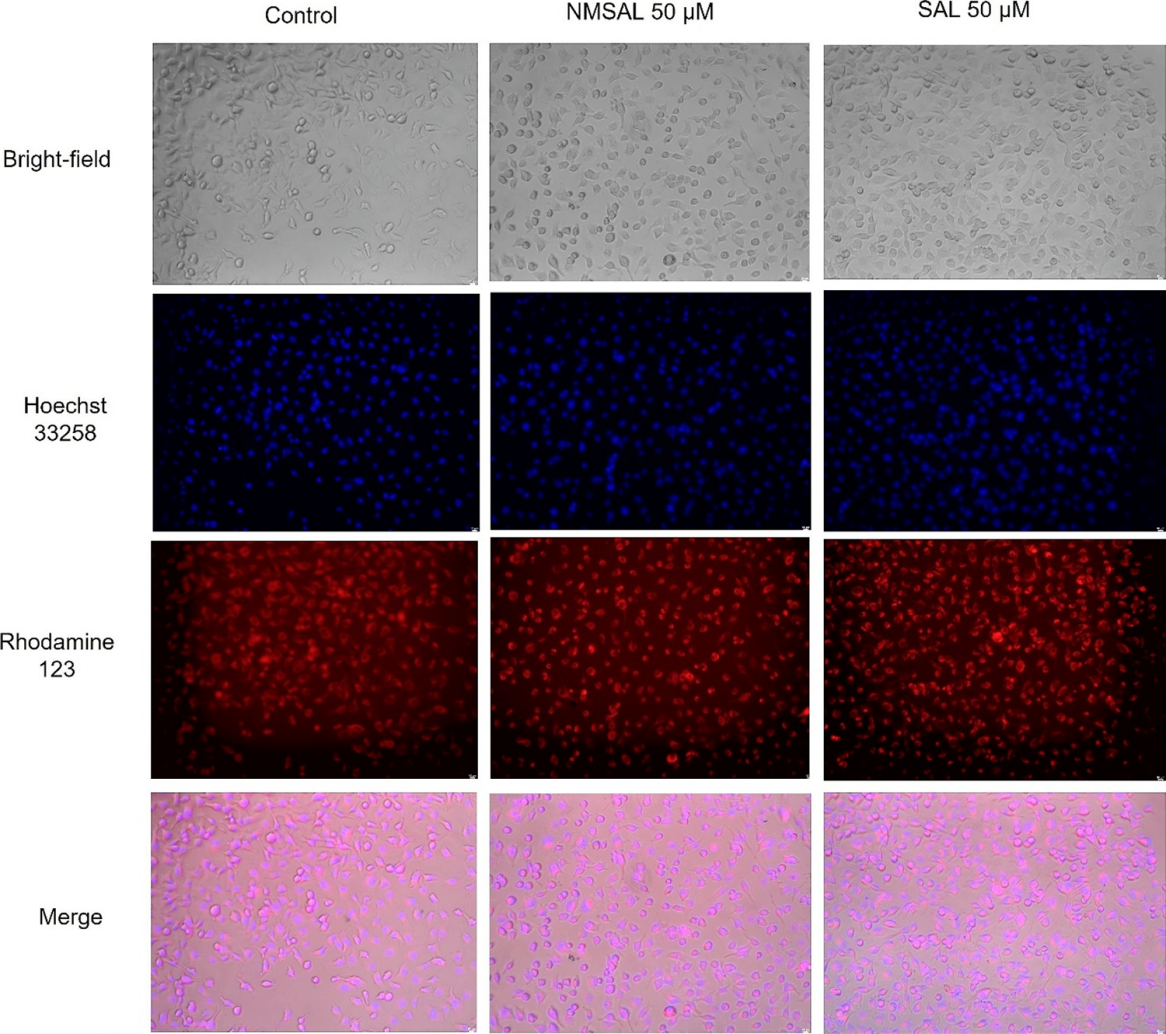
Microscopy images of SH-SY5Y cells exposed for 48 h on 50 μM NMSAL and the reference (50) μM (*R,S*)-SAL. Modified Eagle’s medium with 10% fetal bovine serum was used as the control. Representative pictures were taken by a Leica DMi8 fluorescence microscope Leica DMi8 (100×). No toxic effect on cell morphology confirmed.

**Figure 6:**
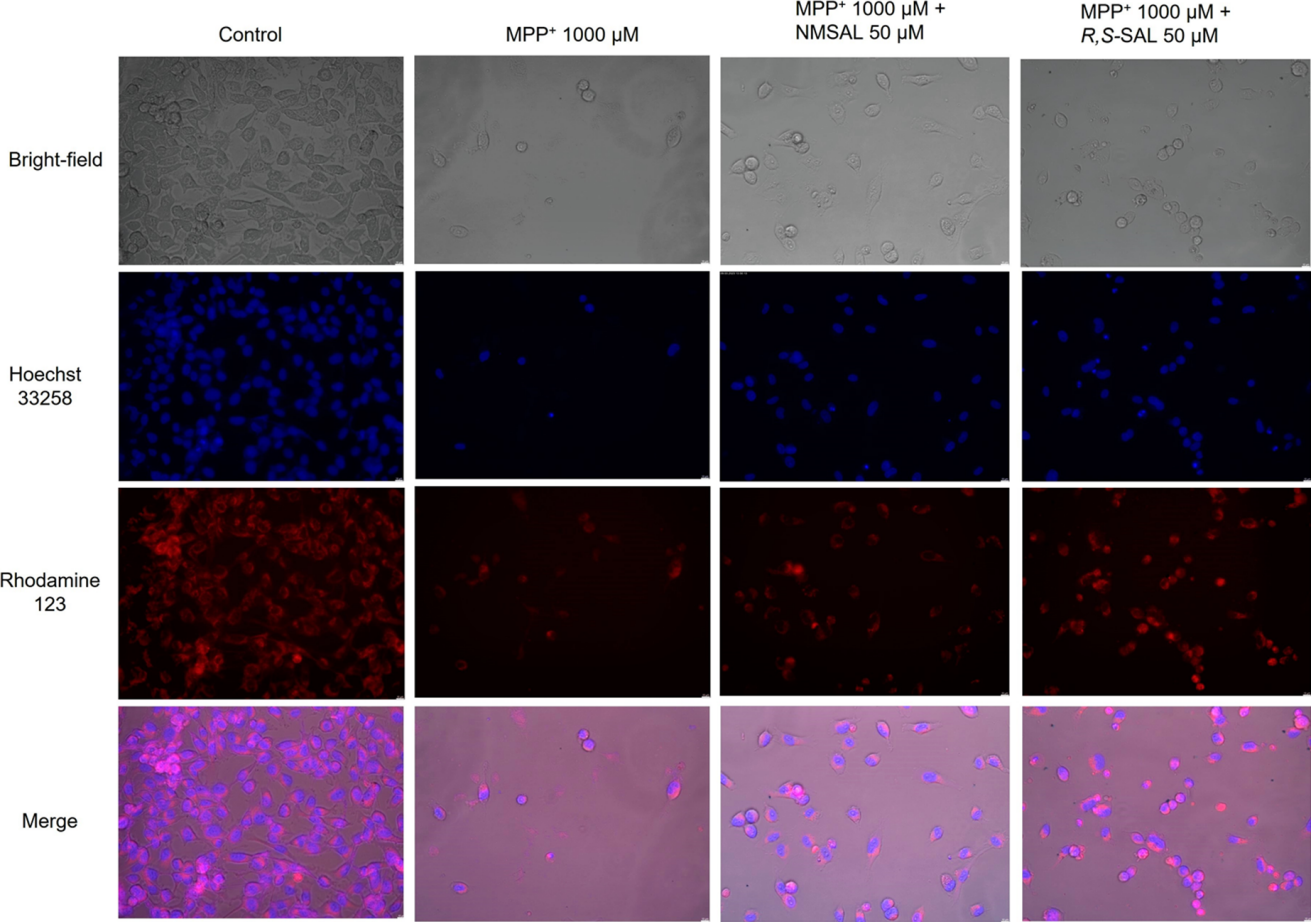
Microscopy images of SH-SY5Y cells exposed for 48 h on 1000 μM MPP^+^ and 1000 μM MPP^+^ together with NMSAL and (*R,S*)-SAL (both in 50 μM). Modified Eagle’s medium with 10% fetal bovine serum was used as the control. Representative pictures were taken by a fluorescence microscope Leica DMi8 (200×).

What is more, photomicrographs of SH-SY5Y human neuroblastoma cells treated with MPP^+^ alone or with MPP^+^ and (*R*), (*S*), and (*R,S*)-SAL or NMSAL, and stained with Hoechst 33258 (a nuclear stain, which emits fluorescence when bound to dsDNA) and rhodamine 123 (a cationic fluorescent dye, which is easily sequestered by active mitochondria without exerting any cytotoxic effects) are shown in Figures 4, 5, and 6, to further support results from MTS studies.

### Molecular Docking of (*R*)- and (*S*)-Salsolinol to D2 Dopamine Receptors

**Figure 7:**
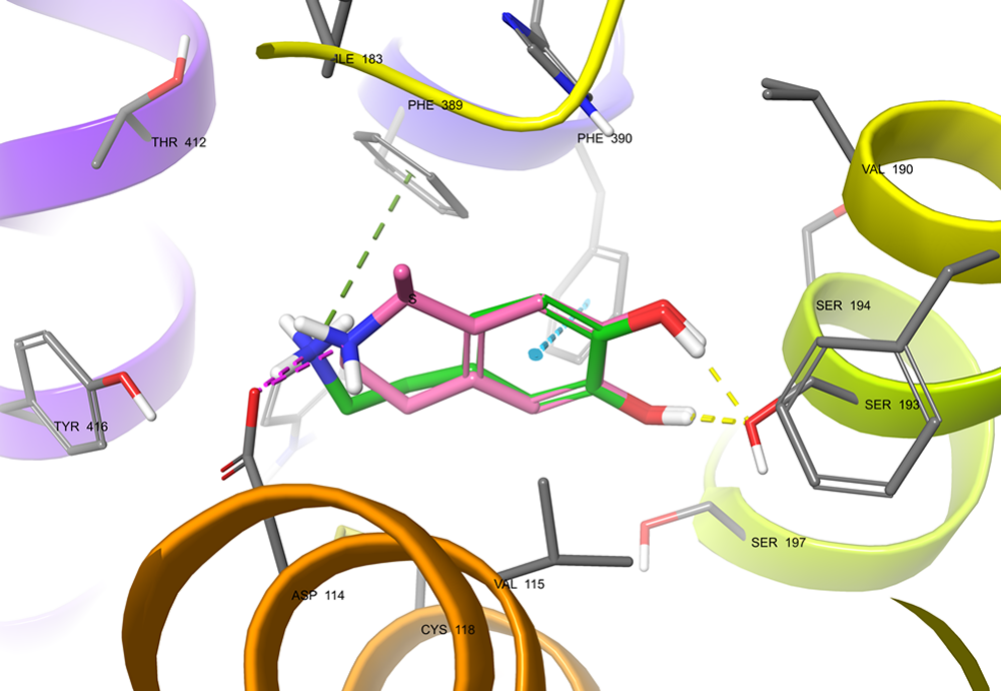
Predicted binding mode of (*S*)-SAL (purple) vs DA (green) in the binding site of dopamine D2 receptor. (*S*)-SAL retained the interactions characteristic of DA – with Asp106 (3.32) (salt bridge/charge-assisted hydrogen bond) and Phe390 (6.52) (CH−π stacking) and a hydrogen bond with Ser193 (5.42) in the orthosteric binding site. Amino acid residues engaged in ligand binding (within 4 Å from the ligand atoms) are represented as thick sticks.

**Figure 8:**
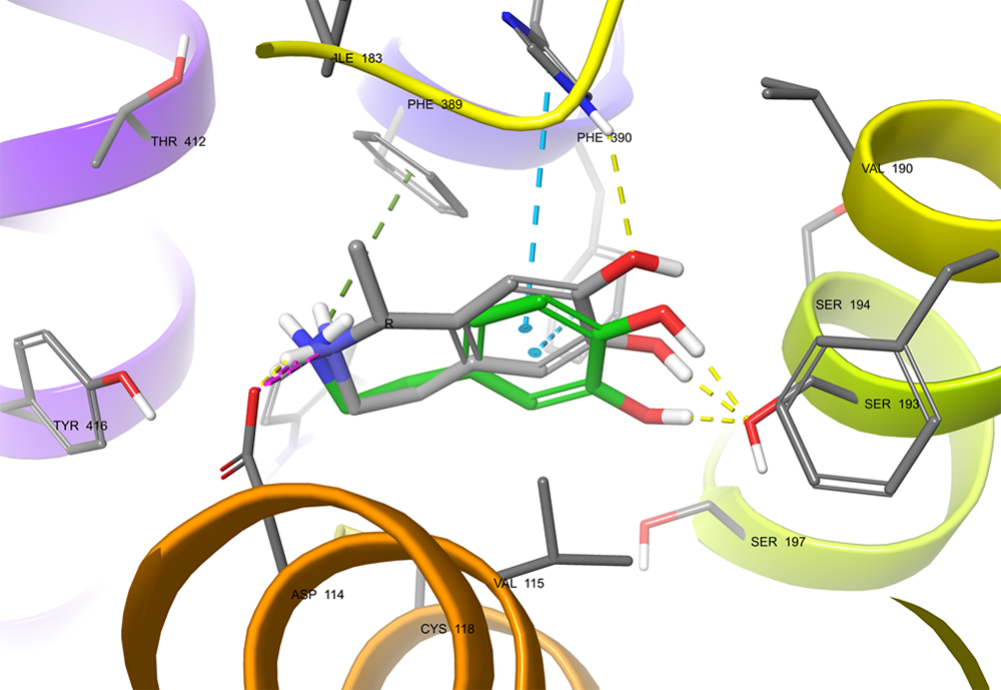
Predicted binding mode of (*R*)-SAL (gray) vs DA (green) in the binding site of dopamine D2 receptor. (*R*)-SAL tends to interact with His393 (6.55) rather than with Phe390 (6.52) (CH−π stacking and H-bond), in addition to other characteristic interactions for dopamine receptor ligands. Amino acid residues engaged in ligand binding (within 4 Å from the ligand atoms) are represented as thick sticks.

In silico analysis confirmed the ability of SAL to interact with dopamine D2 receptors. The *S*-enantiomer arranges in the orthosteric binding site like DA (with a slight difference in the position of the basic nitrogen atom, which should not significantly affect its binding affinity) and makes the same interactions as follows: the salt bridge with Asp114 (3.32) residue together with the π-aromatic stacking with Phe390 (6.52) residue and hydrogen bonds with Ser193 (5.42), which are crucial for binding to this receptor and are characteristic of monoaminergic receptor agonists (Figure 7), such as DA or noradrenaline. The value of the gscore evaluation function for DA is −7.834, while it is −7.692 and −7.554 for (*S*)- and (*R*)-SAL, respectively, indicating a comparable predicted binding energy. Yet, the docking studies of the *R* enantiomer of SAL suggest a slightly different binding mode, showing π-aromatic interaction with His393 (6.55) instead of Phe390 (6.52), which may imply possibly different properties such as receptor affinity or functional activity (Figure 8). To explain this phenomenon and determine a clear binding interaction pattern, we performed a molecular dynamics study. The results indicate first that (*S*)-SAL forms stable interactions in the binding site and maintains its position throughout the 200 ns simulation. The ligand RMSD fluctuations do not exceed 2 Å, indicating that it is not prone to diffuse away from its initial position (Figure S2A). The key interactions, which anchor the molecule in the cavity and presumably affect its functional activity, are maintained for approximately 100% of the simulation time. This applies to H-bonds with Asp114 (3.32) and Ser193 (5.42), the former interaction being reinforced by an ionic bond. When it comes to the aromatic interaction of the catechol ring, contact with Phe390 (6.52) lasted for 30% of the simulation time, being displaced by the interaction with His393(6.55)−π-stacking (47%) and H-bond with the hydroxy group (63%) (Figure S2B). From the pharmacodynamic point of view, the potential of SAL to be a good DA receptor ligand is predicted, yet its pharmacokinetic properties raise a potential concern–the catechol moiety could be readily metabolized in the periphery by COMT, which together with the large number of hydrogen-bond donors may hinder its penetration into the central nervous system (although the prediction in SwissADME Software is favorable in this regard, Table S1 and Figure S1).

**Figure 9:**
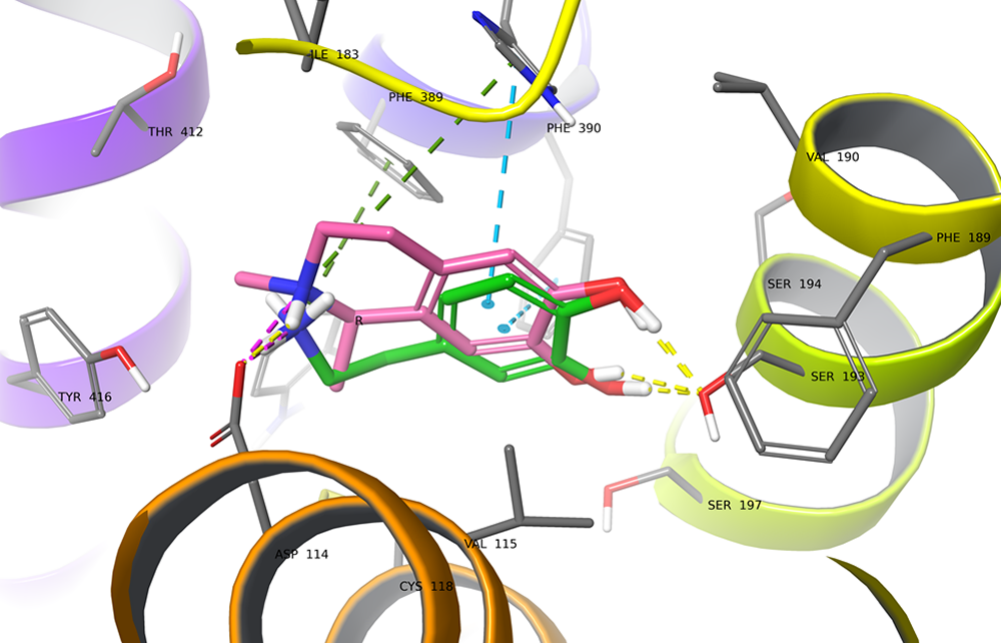
Predicted binding mode of (*R*)-NMSAL (pink) vs DA (green) in the binding site of dopamine D2 receptor. The methyl substituent in the ring portion in best scored pose is directed toward the intracellular part of the binding site and NMSAL interacts with His393 (6.55), which may account for the lower affinity comparing to the desmethyl analogue. Amino acid residues engaged in ligand binding (within 4 Å from the ligand atoms) are represented as thick sticks.

NMSAL may exhibit weaker binding affinity than nonmethyl (*R*)-SAL because a conformation with the methyl group toward the intracellular part is forced here (such a conformation in the case of SAL was scored a worse gscore = −7.037 vs −7.554). NMSAL also interacts with histidine, unlike (*S*)-SAL and DA (Figure 9).

## Discussion

Our results revealed that neither racemic SAL nor its purified enantiomers (obtained from commercially available racemic mixture by means of high-performance liquid chromatography) were toxic to SH-SY5Y human neuroblastoma cells at the concentration of 50 μM, which remains in line with regard to racemic SAL.^23,24^ Interestingly, cell viability in samples treated by MPP^+^ (1000 μM) together with racemic SAL or enantiomers, all used in the dose of 50 μM, was statistically different from SH-SY5Y human neuroblastoma cells treated with MPP^+^ alone (Figure 1B). The MTS assay used for the assessment is in general characterized by good repeatability and due to the fact that MTS produces dark formazan products, the absorbance value range is more sensitive and accurate, especially in comparison with MTT (3-(4,5-dimethylthiazol-2-yl)-2,5-diphenyltetrazolium bromide) assay.^30^ Thus, our results imply the neuroprotective properties of racemic SAL and its enantiomers toward SH-SY5Y human neuroblastoma cell line. Photomicrographs further confirm, complement our previous studies reporting reduction in reactive oxygen species production^23^ and improvement of mitochondrial membrane potential,^24^ and display such properties in vitro (Figure 4). However, no significant difference in the neuroprotective potency between the racemate and the enantiomers was observed in our in vitro experiments. Theoretically, the catechol moiety, which is usually associated with antioxidant properties,^21,22^ could be partially responsible for those neuroprotective properties of SAL as well as its ability of to interact with dopamine D2 receptors. Conversely, Takahashi et al. reported that catechol isoquinolines were more toxic than isoquinolines without catechol structure.^31^ In fact, previously, the majority of studies stressed the cytotoxic effects of racemic SAL to dopaminergic SH-SY5Y human neuroblastoma cells, suggesting its involvement in neuronal cell death in PD and stressed the need to search for mechanism responsible for such neurotoxic action.^31−39^ According to Wang et al., 500 μM of racemic SAL after 24 h of incubation was needed to inhibit SH-SY5Y cells viability by 47.5% assayed by MTT,^32^ while according to both Qualls et al. and Brown et al., 400 μM of racemic SAL was needed, using the same assay and commercially available racemic SAL.^34,35^ Takahashi et al. reported IC_50_ of 540.2 and 296.6 μM for (*R*)- and (*S*)-SAL, respectively, assessed by the Almar Blue assay after 12 h of incubation with SAL enantiomers synthesized according to Teitel et al.^31^ Interestingly, incubation with racemic SAL (500 μM) caused 49.08% ± 1.8 and 22.5% ± 4.5 cell death in undifferentiated and differentiated (by the use of retinoic acid) SH-SY5Y neuroblastoma cells, respectively. The viability of racemic SAL-treated SH-SY5Y cells in medium containing galactose, which is used to deprive of glucose and to improve the responsiveness of the cell cultures to neurotoxins, was 76%.^36^ Pharmacologically, DA has different affinities toward dopamine receptors exerting either immunosuppressive or pro-inflammatory roles depending on its tissue concentrations, reviewed in ref (40). Clearly, in vitro, SAL also exerts its biological action in a dose-dependent manner, and thus, one could ask two fundamental questions. Do the higher (neurotoxic) concentrations of SAL used for in vitro studies reflect in vivo exposure conditions? Are there any direct in vivo data suggesting the involvement of SAL as an endogenous neurotoxin? Due to the fact that SAL is a DA derivative, the highest basal levels were noted in humans in substantia nigra, reviewed in ref (9), up to 204.8 and 213.2 ng/g for (*R*)- and (*S*)-SAL, respectively.^8^ In fact, due to such low concentrations, the measurements of SAL in brain samples have been a real challenge.^9^ And bearing in mind the number (423,796 × 10^3^) and volume (mean 12,922 μm^3^) of pigmented neurons in that region of human brain,^41^ neuronal exposure to locally produced SAL should rather not exceed the neuroprotective doses we used in our in vitro experiments. What is more, previous reports regarding the presence of SAL in CSF fluid^11,13,14^ or urine^5^ of PD patients (already taking levodopa) are only indicative of its involvement in an imbalanced metabolic pathways of DA but evidently should not imply any causation, i.e., PD-related neurotoxicity and/or neurodegeneration. In fact, (*R*)- and (*S*)-SAL were reported to be elevated in urine of healthy humans after 7-day long levodopa treatment.^42,43^ Similarly, liquid diet containing 6.6% ethanol daily supplemented with levodopa (500 μg per rat) for 13 weeks increased striatal levels of SAL in laboratory rodents.^44^ However, an analysis of endogenous SAL enantiomers and DA levels in CSF of untreated denovo PD patients and age- and sex-matched healthy controls showed no significant differences; DA levels did not correlate with (*R*)- or (*S*)-SAL in either group. DA levels did not correlate with either (*R*)- or (*S*)-SAL in both groups.^15^ Plasma levels of (*R*)- and (*S*)-SAL, and DA in untreated denovo PD patients and age- and sex-matched healthy controls (after fasting for 10 h to avoid any nutritional influence) were not significantly different. Yet interestingly, (*R*)-SAL plasma levels were reported to be inversely related to the disease duration and advancement (scored with the Unified Parkinson’s Disease Rating Scale) of PD.^45^

NMSAL was found to accumulate selectively in the human nigrostriatum^6^ and to produce significant behavioral abnormalities in rats after single injection and continuous infusion into striatum.^20,46^ NMSAL was also reported as the only catechol isoquinoline with the ability to deplete dopaminergic neurons in the substantia nigra.^17^ Yet in vitro, we observed no toxic effect of NMSAL on SH-SY5Y neuroblastoma cell viability (assessed by MTS test) up to 750 μM (with IC50 values 864 μM) after 48 h of incubation. What is more, we observed neuroprotective effect of commercially available NMSAL (50 μM) on SH-SY5Y neuroblastoma cell viability damaged by MPP^+^ (1000 μM) after 48 h of incubation (Figure 3). Photomicrographs also display such properties in vitro (Figure 4). Takahashi et al. reported IC_50_ of 581.5 μM of NMSAL, assessed by the Almar Blue assay after 12 h of incubation^31^ and according to Arshad et al. 750 μM of NMSAL induced 50% cell death in MTT assay after 24 h of incubation.^33^ NMSAL was reported to be nonenzymatically oxidized with the formation of hydroxyl radicals^20^ and NMSAL was found to induce DNA damage (determined by detection of DNA damage using a single-cell gel electrophoresis assay in human dopaminergic neuroblastoma SH-SY5Y cells) in comparison with both enantiomers of SAL and 1,2-dimethyl-6,7-dihydroxyisoquinolinium ions. The authors also explained their intentional discrepancy between much higher NMSAL concentrations (up to mM) used for in vitro studies and much lower NMSAL concentrations (∼100 nM) in the substantia nigra measured in control samples from in vivo studies by the following facts: (1) other than dopaminergic and non-neuronal cells in brain samples are present in vivo, (2) about 3% of NMSAL was found to be taken up in the cells, and (3) 100 μM NMSAL could induce DNA damage in 5% of the total neuroblastoma SH-SY5Y cells in vitro.^47^ On the striatal neutral (*R*)-salsolinol *N*-methyltransferase needed for (*R*)-SAL methylation has not been isolated from the human brain or pharmacologically characterized. Consequently, an assumption of the direct involvement of endogenous NMSAL in the PD pathogenesis was made based on increased concentrations of NMSAL in CSF of PD patients and high enzymatic activity of lymphocytes from PD patients,^48^ and thus again based on correlations only. DeCuypere et al. also reported higher ratio of (*R*)-, (*S*)-SAL, and NMSAL vs dopamine in substantia nigra (and interestingly, hippocampus) from PD patients in comparison with healthy human brain samples, yet again no information was given regarding the treatment of those patients.^8^ On the other hand, increased levels of isoquinoline derivatives present following alcohol consumption, such as SAL,^49^ due to acetaldehyde production needed for their synthesis, are not associated with an increased risk of PD development.^50^ What is more, higher urinary concentrations of SAL and NMSAL were found in Tourette syndrome and Tourette syndrome with ADHD patients (yet not de novo)^51^ as well as in adolescents with ADHD only^52^ compared with healthy controls. Thus, such increased levels of SAL and its derivatives might be a consequence of impaired dopaminergic pathways but not an underlying and direct cause of neurodegeneration.

Unarguably, in vivo, the role of SAL is much more complex, especially bearing in mind its uncertain ability to cross the blood brain barrier, discussed in refs (25,53), although the prediction in SwissADME Software is favorable in this regard for both SAL and NMSAL (Figure S1). Indeed, Quintanilla et al. demonstrated that SAL levels were detected in striatum after its systemic administration.^54^ SAL has a ring-closed amine with the p*K*_a_ (the dissociation constant) of 8.49 and thus should be more than in 90% ionized (protonated) at physiological pH,^55^ which may negatively affect membrane permeability, but on the other hand is required for binding to G protein-coupled receptors. Our molecular docking studies revealed that (*S*)-SAL should interact (most probably as an agonist) with dopamine receptors in a similar manner to DA. It should interact with all significant residues essential for binding to the human D2 receptor, via electrostatic interactions between the cationic amino group and Asp-114 in transmembrane spanning region (TM)3, a hydrogen bond between the catechol hydroxyl groups and Ser193 in TM5, and hydrophobic interactions between the phenyl ring and hydrophobic residues such as Phe390 in TM6.^56−58^ (*R*)-SAL should bind differently, via His393 in TM6 (in a similar fashion to class II antagonists^56^), which might suggest different and/or worse affinity or functional activity toward D2 receptor (and other dopamine receptors as well), while NMSAL was found to exhibit even weaker binding in silico. Previously, ex vivo and in vivo studies showed that racemic SAL (1 nM–100 μM) did not displace [3H]SCH-23390 (5 μM, D1 receptor antagonist with minimal effects on the D2 receptor) or [3H]spiperone (10 μM; D2 receptor antagonist) from the striatal membrane preparations.^28^ A single ip injection of SAL (10 mg/kg) inhibited the motor stimulation induced by amphetamine (3 mg/kg ip), which might suggest its antidopaminergic potential.^27^ Such behavioral effects of SAL could not be attributed to its action on DA metabolic pathways either as it was also shown that single administration of racemic SAL did not change the rate of DA metabolism.^28^ The results could be also explained by the fact that apomorphine (0.25 mg/kg given subcutaneously; nonselective DA agonist) in rats pretreated with SAL (100 mg/kg given intraperitoneally) did not induce any behavioral changes (hyperactivity or stereotypy), reversed apomorphine-induced decrease in homovanilic acid (HVA) concentration and SAL displaced [3H]apomorphine from its binding sites with potency similar to DA (with EC_50_ = 225 nM for DA, EC_50_ = 950 nM for racemic SAL). At the same time, pretreatment with racemic SAL (100 mg/kg ip) did not change the cataleptic effect of haloperidol (1 mg/kg ip; inverse D2 agonist and silent D1 antagonist) and did not influence on haloperidol-induced increase in striatal DA metabolism, i.e., an increase in DOPAC and HVA.^29^ Upon prolonged exposure, racemic SAL (intraperitoneal administration of 100 mg/kg of SAL for 14 days) did not affect DA metabolism or its striatal concentration or tyrosine hydroxylase protein level in the rat substantia nigra and striatum^26^ or caused a decrease in DA metabolism in the striatum and substantia nigra.^28^ Melzig et al. reported that (*S*)-SAL bound to dopamine receptors with lower affinity compared to DA, showing the highest affinity to D3 receptor (with *K*_i_ values at 4.79 ± 1.8 and 0.48 ± 0.09 μM toward D2 and D3 receptor, respectively, and above 100 μM for (*R*)-SAL) based on receptor binding analysis using [3H]SCH 23390 (2 nM), [3H]spiperone (0.2 nM), [3H]YM-09151-1 (0.2 nM; D2 and D3 receptor antagonist). Yet, determination of cellular cAMP content revealed that (*S*)-SAL should foremostly act as an agonist of D2-like receptors, thereby inhibiting the basal cAMP production.^59^ Those results correspond well with our molecular docking analysis. Thus, theoretically, only *S*-SAL, similar to other known D2 agonists, could be expected to decrease DA synthesis, release, and signaling upon prolonged exposure. In fact, DA can be both neurotoxic and neuroprotective, and neuroprotection is mediated via D2 receptor.^60^ It was reported that high DA concentrations should have deleterious effects on mitochondrial function such as a decrease in mitochondrial respiration and depolarization of mitochondrial membrane.^61^ What is more, D2 receptor activation was also reported to block the opening of the mitochondrial permeability transition pore and the release of pro-apoptotic signaling molecules^62^ as well as to play a critical role in the rescue of dopaminergic neurons from MPP^+^-mediated degeneration.^63^ And D2 receptor stimulation was already reported to be neuroprotective on mitochondrial function via the inhibition of cAMP/PKA intracellular pathway in G2019S KI Lrrk2 mice.^64^ Unfortunately, it remains unknown whether SAL enantiomers, as DA derivatives, could actively fine-tune/alter such signaling pathways under certain circumstances, i.e., excessive synaptic availability of DA or levodopa. Berríos-Cárcamo et al. also demonstrated that SAL could bind and activate μ-opioid receptors. This pharmacological action could result in the activation of dopaminergic neurons in the mesocorticolimbic system.^65^

Still, some careful consideration should be given to interpretations of the current study since only in vitro and in silico analyses have been performed. Up to date, the results of several failed neuroprotection trials have numerously highlighted the limitations of transferring results from not only cell cultures but also animal models to human physiology and pathology. The use of only one type of cell culture further limits the interpretation. However, it should also be reminded that the presence of dopaminergic neurons is not limited to the central nervous system, and D2 receptors are readily expressed in enteric neurons.^66^ According to Peng et al., selective ablation of D2 receptor in the intestinal epithelium caused more severe loss of dopaminergic neurons in the substantia nigra following MPTP challenge and was accompanied by a reduced abundance of succinate-producing *Alleoprevotella* in the gut.^67^ A wider perspective should be also given to interpretations of our study knowing that SAL is present in common edibles, reviewed in refs (25,40,53) and intestinal microflora.^68^ Recently, a targeted metabolomic analysis revealed that cardiometabolic health benefits related to berry intake might be associated with an increased consumption of SAL and 4-methylcatechol.^69^

## Conclusions

Our study provides in vitro evidence for the neuroprotective potential of both enantiomers of salsolinol as well *N*-methyl-(*R*)-salsolinol. We believe our results highlight the need to acknowledge salsolinol as a biologically active and meaningful dopamine derivative/metabolite and to further explore the neuroregulatory role of enantiomers of salsolinol in the central and peripheral nervous system. What is more, our molecular docking analysis showed that stereoisomers of salsolinol should exhibit distinct (possibly opposite) ability to interact with dopamine D2 receptors, which could explain tentative results of previously published studies with the use of a racemic mixture. Thus, bearing in mind possible exogenous and endogenous origins of enantiomers of salsolinol, future studies should determine their role in the context of levodopa-induced dyskinesias and neuroprotection in the first instance.

## Materials and Methods

### Separation and Purification of (*R*) and (*S*)-SAL

(*R*)- and (*S*)-SAL were separated by means of high-performance liquid chromatography (HPLC) from commercially available racemic SAL (*(R*/*S*)-salsolinol), as described previously with some modifications.^70^ Briefly, a solution of racemic SAL hydrochloride (9.9 × 10^–3^ M; sc-215838, Santa Cruz Biotechnology, Santa Cruz, CA, USA) was prepared in water. Then, 50 μL of this solution was injected onto an HPLC system equipped with a NUCLEODEX β-cyclodextrin-modified column 200 × 8 mm i.d. (Macherey-Nagel, Germany) kept at 30 °C; an isocratic pump adjusted to 0.80 mL/min (Shimadzu LC-10AD, Kyoto, Japan); and an LC-4C BAS amperometric detector set at a potential of 0.7 V. The mobile phase was 100 mM ammonium acetate containing 10 mM triethylamine (pH 4.0). Under these conditions, it was reported that (*S*)-SAL was the first to elute.^54^ Once a racemic SAL sample was injected into the HPLC instrument, the enantiomers were separated according to their retention time ((*S*)-SAL: 17.6 min; (*R*)-SAL: 22 min) and collected after disconnecting the electrochemical detector in order to avoid sample oxidation. The procedure was repeated until obtaining sufficient amounts of the enantiomers. The mobile phase was eliminated by freezing at −80 °C for 4 h and subsequent lyophilization at −50 °C overnight. The samples were stored at −20 °C in amber microtubes.

A series of 8 samples of racemic SAL were injected into the HPLC, and its *S* and *R* enantiomers were purified according to their retention time. For (*S*)-SAL, 5 mL of eluate was obtained. For (*R*)-SAL 10 mL of eluate were obtained. The comparison of the areas showed that the eluate contained (*S*)-SAL with less than 0.1% of (*R*)-SAL. By comparison with the peak areas obtained for racemic SAL standards, we found that the eluate of (*S*)-SAL had a concentration of approximately 2.4 × 10^–4^ M. The comparison of the areas showed that the eluate contained (*R*)-SAL with about 4% of (*S*)-SAL. By comparison with the peak areas obtained for racemic SAL standards, we found that the eluate of (*R*)-SAL has a concentration of approximately 2.9 × 10^–4^ M. The eluate for (*S*)-SAL was divided into 5 aliquots of 1 mL in dark microtubes. The eluate for (*R*)-SAL was divided into 6 aliquots of 1.5 mL and 1 aliquot of 1 mL in dark microtubes. The samples were frozen during 4 h at −80 °C and then lyophilized (−50 °C) overnight. To further check the amount of the purified enantiomers, the lyophilized product obtained from the 1 mL aliquot of the (*R*)-SAL eluate was dissolved into 100 μL of water. A calibration curve of (*R,S*)-SAL vs absorbance at 290 nm was obtained. The spectrophotometric determination of the concentration of the solution obtained from lyophilized (*R*)-SAL (1 mL) indicated a value of 1.64 mM.

### In Vitro Experiments

#### Cell Culture and Reagents

SH-SY5Y (ATCC CRL-2266TM) neuroblastoma cell line was purchased from ATCC (American Type Culture Collection, USA). Undifferentiated SH-SY5Y cells are considered to be most reminiscent of immature catecholaminergic neurons, they express dopaminergic neuronal markers, and importantly, these cells also express dopamine receptor 2 and 3, making them an excellent in vitro system to study mechanisms of neurotoxicity, reviewed in ref (71). The cells were cultured in modified Eagle’s medium (MEM) with 10% fetal bovine serum (FBS) obtained both from Gibco (USA). In all experiments, MEM supplemented with 10% FBS was used as the control. (*R,S*)-salsolinol (SAL) of purity ≥99% was purchased from Cayman Chemical (USA). *N*-Methyl-(*R*)-salsolinol (NMSAL) of purity ≥95% was purchased from Santa Cruz (USA). MPP^+^ (1-methyl-4-phenylpyridinium iodide) of purity ≥98% was purchased from Angene (India). The CellTiter 96 AQueous Non-Radioactive Cell Proliferation Assay with a novel tetrazolium compound, 3-(4,5-dimethylthiazol-2-yl)-5-(3-carboxymethoxyphenyl)-2-(4-sulfophenyl)-2*H*-tetrazolium (MTS), was purchased from Promega (USA). Rhodamine 123 and Hoechst 33258 were purchased from Sigma-Aldrich (Merck LifeScience, Poland) and ThermoFisher Scientific (USA), respectively.

#### MTS Assay

Part 1: SH-SY5Y CRL-2266 (American Type Culture Collection, USA) cells were seeded in 96-well plate at a concentration of 0.7 × 10^4^ cells/well in 100 μL culture medium and cultured for 24 h at 37 °C in an atmosphere containing 5% of CO_2_ to reach 70% confluence. For the toxicity evaluation, cells were incubated with (*R*), (*S*), or (*R,S*)-SAL at the final concentration 50 μM for 48 h. For the neuroprotection assessment, cells were preincubated first for 1 h with (*R*), (*S*), or (*R,S*)*-*SAL at the final concentration 50 μM and next MPP^+^ (1000 μM final concentration) was added.

Part 2: SH-SY5Y cells were seeded in 96-well plate at a concentration of 0.7 × 10^4^ cells/well in 100 μL culture medium and cultured for 24 h at 37 °C in an atmosphere containing 5% of CO_2_ to reach 70% confluence. For the toxicity evaluation, cells were incubated with NMSAL for 48h at the following concentrations: 3000, 2000, 1500, 1000, 750, 500, 375, 180, 93, 40, 23, 11, and 5.80 μM. For the neuroprotection assessment, cells were preincubated first for 1 h with NMSAL or (*R,S*)-SAL at the final concentrations 50 μM, and next MPP^+^ (1000 μM final concentration) was added.

After 48 h of incubation, both in part 1 and 2 of the experiment, the MTS labeling mixture was added to each well, and cells were incubated under the same conditions for 5 h. The absorbance was measured by using a microplate reader EnSpire (PerkinElmer, USA) at 490 nm. All measurements were performed in quadruplicate and were analyzed by one way ANOVA followed by Bonferroni’s comparison test (GraphPad Prism 8.0). Final data, in both Part 1 and Part 2, were pooled from two independent experiments.

#### Fluorescence Microscopy

SH-SY5Y cells were seeded and incubated with (*R*), (*S*), or (*R,S*)-SAL, NMSAL, and MPP^+^ according to the same procedure as described above. After 48 h of incubation the cells were rinsed with PBS and the mixture containing 10 μM rhodamine 123 and 10 μM Hoechst 33258 was added and incubated at 37 °C and 5% CO_2_ for 30 min. Representative pictures were taken next by a Leica DMi8 fluorescence microscope Leica DMi8.

### In Silico Analysis

Molecular modeling studies were performed using the Small-Molecule Drug Discovery Suite (Schrödinger, Inc.). The structures of DA, SAL, and NMSAL were prepared in protonated forms using LigPrep and docked with Glide XP (H-bond constraint and centroid of a grid box were set at Asp3.32). The previously prepared homology model of human dopamine D2 receptor, optimized in induced-fit procedure with a partial agonist bifeprunox, served as a target protein structure.^72^ Optimal pose selection was based on GlideScore scoring function and qualitative interaction analysis. Molecular dynamics (MD) simulation was performed using the Desmond GPU package. System for MD simulation was prepared using the System Builder module. The complex of (*S*)-SAL obtained in the docking studies was placed in the SPC solvent model and minimized using a Brownian motion simulation (100 ps). The POPC membrane was added to the system, and an appropriate number of counterions maintaining charge neutrality were added. 200 ns simulation using OPLS4 force field was run in *NP*γ*T* ensemble, and trajectories were saved in 20 ps intervals. Simulation interaction protocols were generated to calculate RMSD plots and interaction diagrams.

## Supporting Information Available

Additional experimental details such as computed physicochemical properties and results of molecular dynamics simulation (PDF)

## ABBREVIATIONS

CSF: cerebrospinal fluid
DA: dopamine
IC50: half-maximal inhibitory concentration
MPTP: 1-methyl-4-phenyl-1,2,3,6-tetrahydropyridine
MPP^+^: 1-methyl-4-phenylpyridinium
MTS: 3-(4,5-dimethylthiazol-2-yl)-5-(3-carboxymethoxyphenyl)-2-(4-sulfophenyl)-2H-tetrazolium
MTT: 3-(4,5-dimethylthiazol-2-yl)-2,5-diphenyltetrazolium bromide
PD: Parkinson’s disease
SAL: salsolinol, 1-methyl-1,2,3,4-tetrahydroisoquinoline-6,7-diol
(*R*)-SAL: *R* enantiomer of salsolinol
(*S*)-SAL: *S* enantiomer of salsolinol
NMSAL: *N*-methyl-(*R*)-salsolinol
