# Supplementary material for: Neuroprotective Activity of Enantiomers of Salsolinol and N-Methyl-(R)-salsolinol: In Vitro and In Silico Studies
Source: ACS Omega. 2023 Oct 5;8(41):38566–76. doi: 10.1021/acsomega.3c05527 (PMC10586258; doi:10.1021/acsomega.3c05527)
Supplement: Supplementary file 1 — ao3c05527_si_001.pdf [file ao3c05527_si_001.pdf]

Supporting information

The neuroprotective activity of enantiomers of salsolinol and N-methyl-*(R)*-salsolinol — *in vitro* and *in silico* studies

Magdalena Kurnik-Łucka[1*], Gniewomir Latacz[2], Adam Bucki[3], Mario Rivera-Meza[4], Nadia Khan[1,2], Jahnobi Konwar[2], Kamil Skowron[1], Marcin Kołaczkowski[3], Krzysztof Gil[1]

* Corresponding author

[1]Department of Pathophysiology, Jagiellonian University Medical College, Krakow, Poland

[2]Department of Technology and Biotechnology of Drugs, Jagiellonian University Medical College, Krakow, Poland

[3]Department of Medicinal Chemistry, Jagiellonian University Medical College, Krakow, Poland

[4]Laboratory of Experimental Pharmacology, Faculty of Chemical Sciences and Pharmaceutical Sciences, University of Chile, Santiago, Chile

Table S1. Physicochemical properties, pharmacokinetics, and drug-likeness of *(R/S)*-salsolinol and N-methyl-*(R)*-salsolinol assessed via the SwissADME web tool available at http://www.swissadme.ch/index and accessed on17.04.2023 [1].

	***(R/S)*-salsolinol**	**N-methyl-*(R)*-salsolinol**
Chemical structure	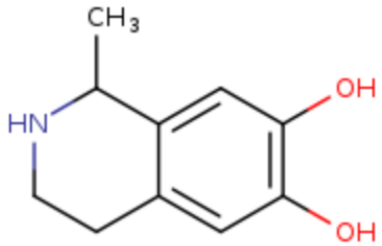	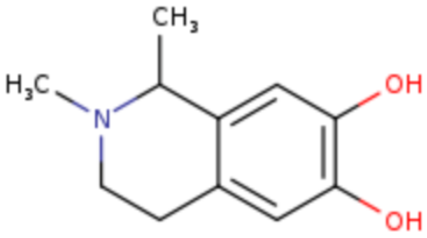
SMILES	CC1C2=CC(=C(C=C2CCN1)O)O	CC1C2=CC(=C(C=C2CCN1C)O)O
Molecular weight	179.22 g/mol	193.24 g/mol
Number of H-bond donors	3	2
Topological Polar Surface Area (TPSA)	52.49 Å^2	43.70 Å^2
Log $P_{o/w}$ (WLogP)	0.60	0.94
Gastrointestinal (GI) absorption	High	
Blood-brain barrier (BBB) permeant	Yes	
P-glycoprotein (P-gp) substrate	No	

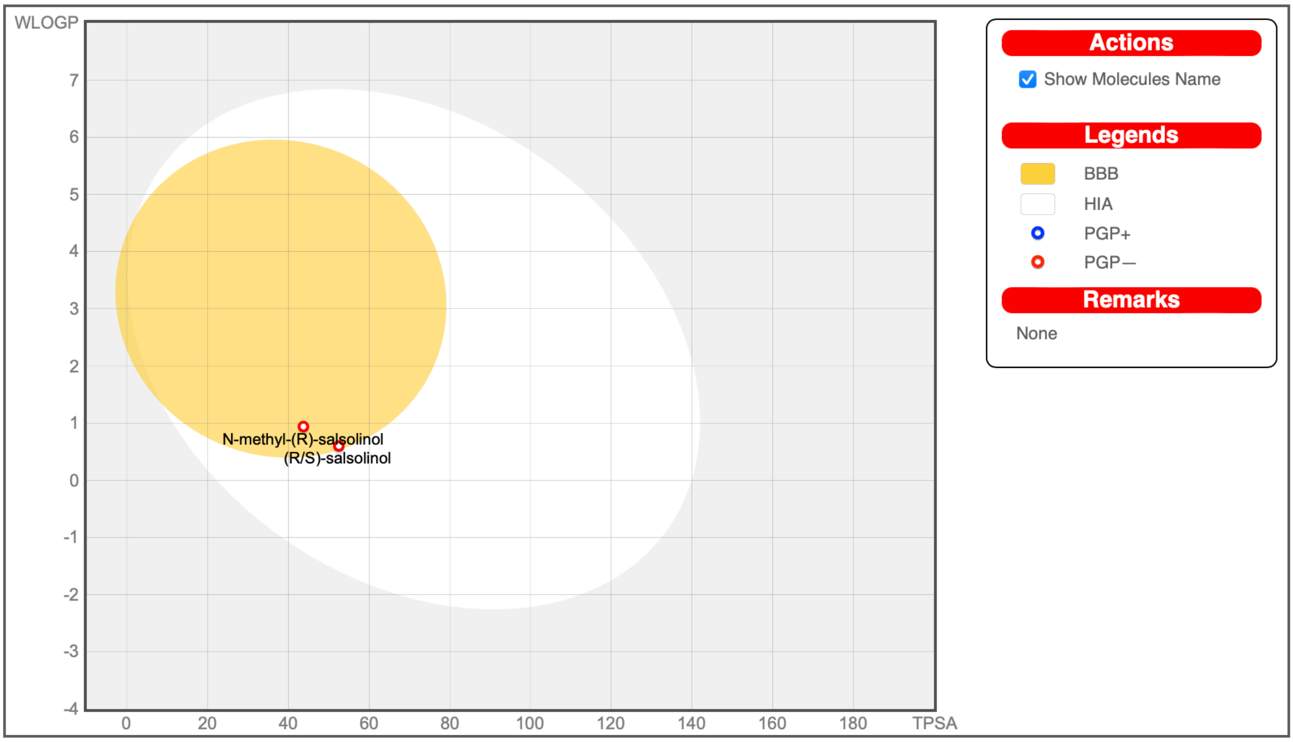

Figure S1. The Brain Or IntestinaL EstimateD permeation method (BOILED-Egg) modeled by computing the lipophilicity and polarity of *(R/S)*-salsolinol and N-methyl-*(R)*-salsolinol to predict their gastrointestinal absorption and brain access, assessed via the SwissADME web tool available at http://www.swissadme.ch/index and accessed on 16.05.2023. The yellow region indicates good penetration across the blood-brain barrier (BBB) [1,2].

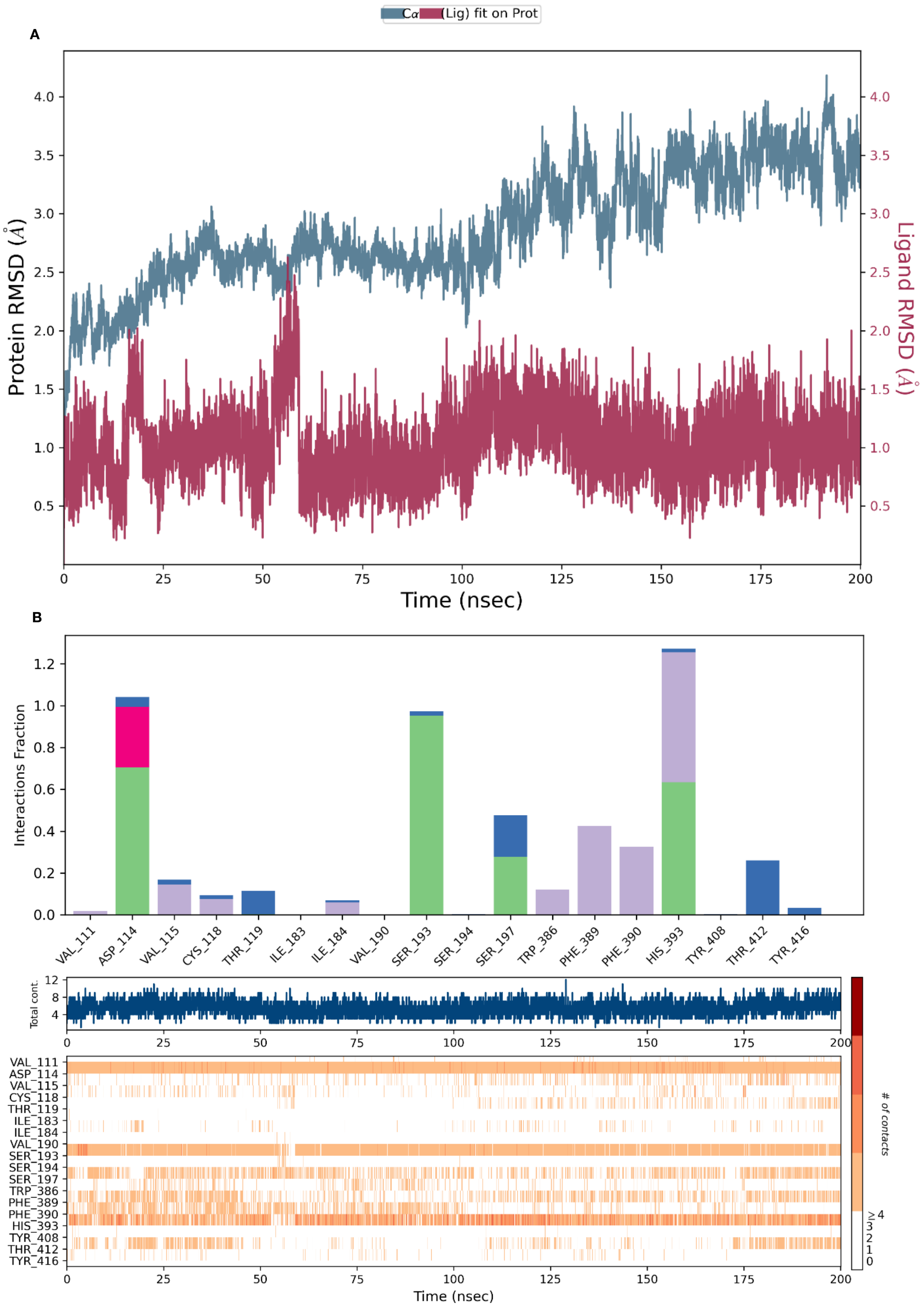
Cα (Lig) fit on Prot
A
Protein RMSD (Å)
Ligand RMSD (Å)
Time (nsec)
B
Interactions Fraction
VAL_111
ASP_114
VAL_115
CYS_118
THR_119
ILE_183
ILE_184
VAL_190
SER_193
SER_194
SER_197
TRP_386
PHE_389
PHE_390
HIS_393
TYR_408
THR_412
TYR_416
Total cont.
# of contacts
≥4
Time (nsec)

Figure S2A and S2B. Molecular dynamics simulation (200 ns) for *(S)*-salsolinol.

References:
1. Daina, A., Michielin, O., Zoete, V. SwissADME: a free web tool to evaluate pharmacokinetics, drug-likeness and medicinal chemistry friendliness of small molecules. *Sci. Rep*. **2017**, 7, 42717. doi: 10.1038/srep42717.

2. Daina A, Zoete V. A BOILED-Egg To Predict Gastrointestinal Absorption and Brain Penetration of Small Molecules. *Chem. Med. Chem*. **2016**, 11, 1117-21. doi: 10.1002/cmdc.201600182.
